# Smoking and reproduction: The oviduct as a target of cigarette smoke

**DOI:** 10.1186/1477-7827-3-52

**Published:** 2005-09-28

**Authors:** Prue Talbot, Karen Riveles

**Affiliations:** 1 Department of Cell Biology and Neuroscience, Interdepartmental Graduate Program in Environmental Toxicology, University of California, Riverside, CA 92521, USA

## Abstract

The oviduct is an exquisitely designed organ that functions in picking-up ovulated oocytes, transporting gametes in opposite directions to the site of fertilization, providing a suitable environment for fertilization and early development, and transporting preimplantation embryos to the uterus. A variety of biological processes can be studied in oviducts making them an excellent model for toxicological studies. This review considers the role of the oviduct in oocyte pick-up and embryo transport and the evidence that chemicals in both mainstream and sidestream cigarette smoke impair these oviductal functions. Epidemiological data have repeatedly shown that women who smoke are at increased risk for a variety of reproductive problems, including ectopic pregnancy, delay to conception, and infertility. In vivo and in vitro studies indicate the oviduct is targeted by smoke components in a manner that could explain some of the epidemiological data. Comparisons between the toxicity of smoke from different types of cigarettes, including harm reduction cigarettes, are discussed, and the chemicals in smoke that impair oviductal functioning are reviewed.

## A. Background

Exposure to cigarette smoke may be either active or passive, and the type of smoke inhaled in each case has a different origin. Mainstream smoke is the smoke that an active smoker inhales with each puff, while sidestream smoke, the main component of environmental tobacco smoke, burns off the end of a lit cigarette and is the smoke inhaled by passive smokers. While the association between inhalation of mainstream smoke and cardiovascular disease and cancer has been established for many years, the impact of smoking on reproduction is recognized, but less well characterized and less well known [1]. Epidemiological studies have repeatedly shown that women of child bearing age who actively inhale mainstream smoke have higher rates of infertility, spontaneous abortion, ectopic pregnancy, tubal infertility, increased time to conception, and intrauterine growth retardation than nonsmokers [2-15]. Increases in infertility and ectopic pregnancy in smokers could be due to impairment of oviductal functioning. In patients with primary tubal infertility, 39% were smokers when they started trying to conceive in contrast to only 16% in the non-smoking group (OR = 2.7) [10]. Heavy smoking (> 5 pack-years) increased the odds ratio to 4.2, and similar dose related effects have been repeatedly observed [11,16].

The realization that sidestream smoke exposure adversely affects human health is even more recent [17]. In 1992, the Environmental Protection Agency published a monograph summarizing evidence that exposure to environmental tobacco smoke can produce adverse effects on cardiovascular and lung health and encouraged broader investigation in this area [17]. Subsequently, a number of studies have addressed the effect of passive smoking on various aspects of human health including reproduction and have concluded that adverse reproductive outcomes, such as delayed time to conception and reduced birth weight, do occur as a consequence of exposure to environmental tobacco smoke during pregnancy [18-30]. Moreover, an *in vitro *fertilization lab recently concluded that while fertilization rates and embryo quality were similar in smokers and non-smokers, implantation and pregnancy rates were adversely affected by both active and passive smoking when compared to non-smoking controls [31].

Recent reviews have addressed issues of cigarette smoke exposure and various facets of reproduction including delayed time to conception, ovarian effects and premature menopause, implantation failure, fetal growth restriction and growth retardation, placental abnormalities, reduced fecundity, congenital abnormalities, and effects on male reproduction [32-34]. However, most prior reviews have not considered smoke's interaction with the oviduct, an organ vital to reproduction. The purpose of this paper is to review the functions of the oviduct, in particular those that involve movement of gametes and embryos, and to evaluate evidence that exposure to mainstream or sidestream cigarette smoke can negatively impact oviductal functioning and thereby adversely affect reproductive outcomes. We will also consider evidence that commercial cigarettes, including harm reduction and light cigarettes, contain toxicants that impair oviductal functioning, and we will discuss the specific chemicals in smoke that impair oviductal functioning. Some of these chemicals adversely affect oviductal processes at extremely low doses, are often considered safe, and are added to cigarettes and other consumer items.

## B. Functions of the oviduct

**Figure 1:**
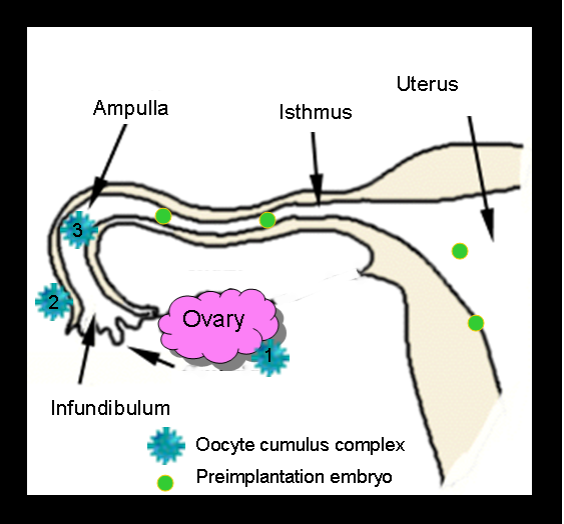
Schematic diagram showing the three anatomical regions of the oviduct (infundibulum, ampulla, and isthmus) and the regions of the oviduct where oocyte cumulus complexes and preimplantation embryos can be found. Oocyte cumulus complexes are ovulated from ovaries (#1), picked-up by the outer surface of the infundibulum (#2), and moved toward the ostium (unlabeled arrow) by ciliary beating then into the ampulla for fertilization (#3). Fertilized eggs and embryos are transported through the isthmus to the uterine cavity where they then can implant in the uterine wall.

The oviduct, which is divided anatomically into the infundibulum, ampulla, and isthmus, plays important roles in mammalian reproduction (Fig. 1) [35-41]. The infundibulum is responsible for picking-up the oocyte cumulus complex following ovulation and moving it into the ampulla where fertilization occurs. Simultaneously, the oviduct moves sperm in the opposite direction from a reservoir near the uterotubal junction toward the ampulla [42]. The oviduct also provides a suitable microenvironment for capacitation of spermatozoa, fertilization, preimplantation development, and transport of the preimplantation embryos to the uterus. The movement of the embryo through the oviduct to the uterus is carefully timed by ovarian hormones and signals from the embryos [43]. While smoke exposure could affect any of these processes, most current evidence links smoke to effects on oocyte cumulus complex pick-up and embryo transport, which will be reviewed in more detail in the following sections.

### (1) Oocyte cumulus complex pick-up by the infundibulum

**Figure 2:**
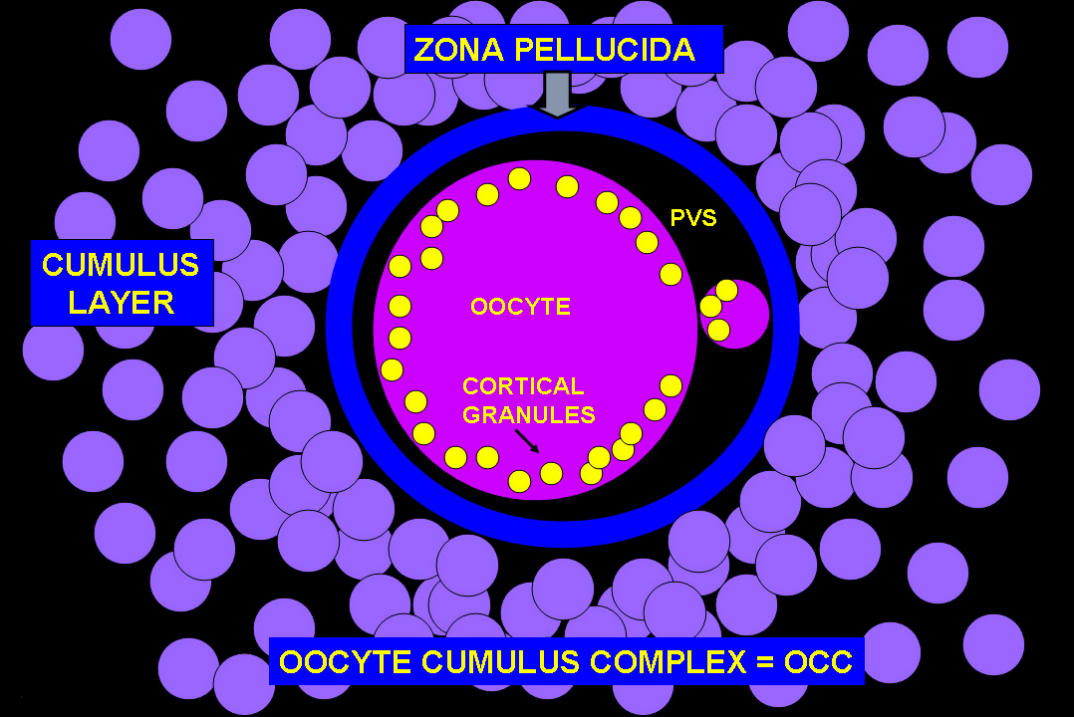
Schematic diagram of an oocyte cumulus complex after ovulation from an ovarian follicle. The oocyte and polar body are contained within the zona pellucida. Immediately outside the zona, cells are densely packed to form the corona radiata outside of which are numerous cumulus cells. The cumulus cells are widely separated from each other by spaces filled with an extracellular matrix (matrix is shown in Figure 5).

The infundibulum is the portion of the oviduct closest to the ovary and is responsible for picking up the oocyte cumulus complex following its ovulation from a mature ovarian follicle [44,45]. The oocyte cumulus complex consists of a centrally located oocyte, which is in turn surrounded by the zona pellucida, corona radiata, and cumulus cells (Fig. 2) [46-48]. The complex contains 5,000–8,000 cumulus cells, depending on the species, and these are separated from each other by an extracellular matrix, which plays an important role in the pick-up process. The structure and distribution of the extracellular matrix between cumulus cells has been well characterized in a number of species including humans [46,49-52]. Biochemically, the matrix is rich in hyaluronan (hyaluronic acid) [53-55], which is cross-linked by inter-alpha trypsin inhibitor [56-58]. TSG-6 (the secreted product of the tumor necrosis factor-stimulated gene 6) also binds to hyaluronan in the cumulus matrix [59-61]. The importance of these matrix components to reproduction is demonstrated by the TSG-6 knockout mouse which fails to assemble a cumulus matrix and is infertile [62].

**Figure 3:**
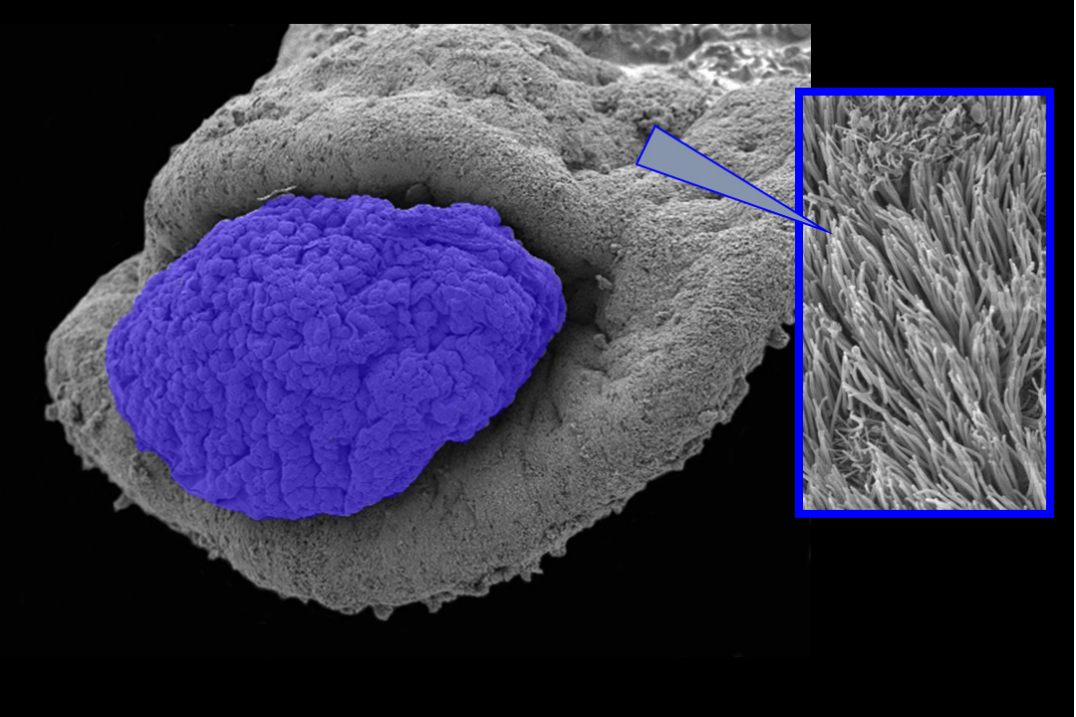
Scanning electron micrograph showing a hamster oocyte cumulus complex, colorized blue, entering the ostium of an infundibulum. The outer and inner surfaces of the infundibulum are covered with cilia (inset).

Oocyte pickup by the infundibulum is a complex process that involves both ciliary beating and adhesion between the oviductal epithelium and the oocyte cumulus complex [63-73]. Both the inner and outer surfaces of the infundibulum are covered with ciliated epithelium (Fig. 3) [74]. Following ovulation, the oocyte cumulus complex travels along the outer surface of the infundibulum and enters the oviduct through the ostium (Fig. 3) [45,75]. The complex then rapidly moves to the ampulla where fertilization occurs. Although infundibular smooth muscle may contract during the pick-up process, it does not appear to be necessary for pick-up, which still occurs when muscle contraction is inhibited with isoproterenol [76].

Huang et al., developed an *in vitro *method for measuring oocyte pickup rate using hamster infundibula [71]. At room temperature, oocyte pickup rate averaged 55.2 + 10.6 um/sec and was dependent on the viscosity of the culture medium and temperature. Moreover, complexes were observed to move along particular pathways on the surface of the infundibula depending on where they were placed. This *in vitro *bioassay has subsequently evolved to allow measurement of smooth muscle contraction [77] and adhesion of the oocyte cumulus complex to the infundibulum [72].

**Figure 4:**
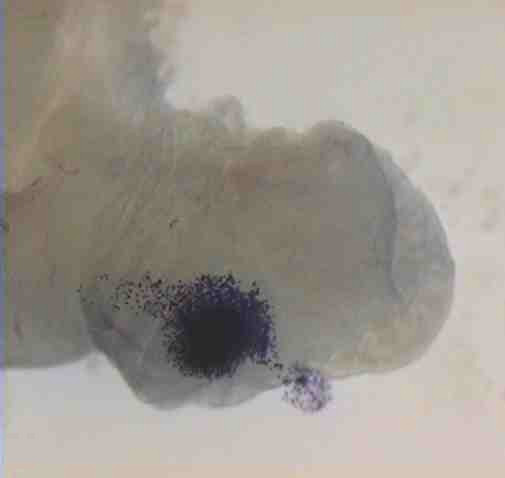
Micrograph showing a hamster oocyte cumulus complex, stained blue, on the outer surface of an infundibulum. Click the link to view a video of this complex being picked up by the oviduct. Reprinted from Molec Biol Cell 10:5–9, 1999 (with permission). See also

The hamster infundibular explant has also been used to analyze the process of pick-up in hamsters in conjunction with video microscopy [45]. While small particles such as *Lycopodium *spores can move over the infundibular surface in the currents created by ciliary beating [45,78], the large mass of the oocyte cumulus complex does not allow it to move in the fluid currents created by ciliary beating alone. In addition to ciliary beating, adhesion between the cumulus cell matrix and the tips of the cilia is necessary to move the complex over the surface of the infundibulum [45,72]. The cumulus matrix attaches the complex to the infundibulum, and as the cilia beat in the direction of the ostium, the oocyte cumulus complex glides over the surface of the infundibulum until it reaches and enters the ostium. Figure 4 (Additional file 1) links to a video showing the movement of a hamster oocyte cumulus complex over the surface of an infundibulum. Additional videos of this process can be viewed at . In hamsters, the oocyte cumulus complex is larger in diameter than the opening of the ostium, and in order for the complex to enter the oviduct, it goes through a "churning" process that compacts the matrix between the cumulus cells making the complex small enough to pass through the ostium [45]. During churning, the oocyte is sometimes squeezed from the center of the complex to the periphery. Pick-up of a human oocyte cumulus complex has been observed *in vivo *using transvaginal hydrolaparascopy and involves adhesion of the complex to the tumescent fimbria of the infundibulum with ciliary beating drawing the complex into the ostium [75].

**Figure 5:**
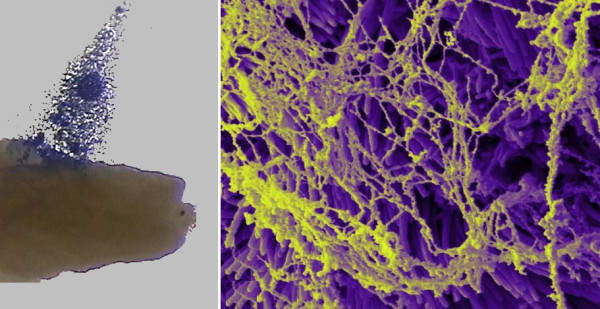
Micrographs showing adhesion between the oocyte cumulus complex and the infundibulum. (A) Stereoscopic micrograph of an oocyte cumulus complex, colorized blue, being pulled off the surface of an infundibulum using forceps. The matrix of the complex adheres to the infundibulum. Complexes do not adhere to most other surfaces. (B) Scanning electron micrograph of cumulus matrix adhering to cilia on the outer surface of an infundibulum. The matrix was left behind by an oocyte cumulus complex that was picked-up by the infundibulum.

Adhesion plays an essential role in the pick-up process (Fig. 5) [66,72]. Oocytes denuded of cumulus cells are not picked up [66], and when matrix is not secreted by the cumulus cells, the complex fails to attach to the infundibulum and it is not moved into the oviduct [72]. Polycationic compounds can block oocyte cumulus complex pick-up apparently by blocking transient adhesion between the tips of the cilia and the complex [67]. Interestingly, peritoneal fluid from women with endometriosis contains a macromolecule (< 100,000 kDa) that when assayed with hamster infundibula *in vitro *coats the cilia on the surface of the infundibula and blocks adhesion and hence pick-up of the human oocyte cumulus complex by the hamster infundibulum [79,80]. Transmission electron microscopy revealed that adhesion during complex pickup occurs specifically between the cumulus matrix and the crowns at the tips of the infundibular cilia [72]. An *in vitro *assay using vacuum from a low flow peristaltic pump has been developed to measure adhesion between the oocyte cumulus complex and infundibulum [72]. This assay was used to show that factors that either increase or decrease adhesion can interfere with the pick-up process. If the matrix of the oocyte cumulus complex is made less sticky by compacting it or treating it with poly-l-lysine, the complex cannot adhere tightly enough to the infundibulum to be successfully picked up [72]. Conversely, if adhesion is increased, for example by treating complexes or the oviduct with the lectin wheat germ agglutinin, ciliary beating is not strong enough to transiently detach the complex and move it to the ostium. Thus successful pick-up requires a delicate balance between proper strength of adhesion of the complex to the infundibulum and ciliary beating towards the ostium.

The ampulla serves as a reservoir for the oocyte cumulus complex, and hormonally controlled oviductal secretions play an important role in creating a suitable microenvironment for fertilization and initial preimplantation development [37,44,81,82]. After entering the female reproductive tract, sperm are stored in a reservoir near the uterotubal junction [42]. As some sperm leave the reservoir and move through the isthmus of the oviduct, they become fully capacitated and their motility becomes hyperactivated [38,83,84]. Hyperactivation is thought to be critical to fertilization as it allows sperm to detach from the oviductal epithelium, move in the lumen of the oviduct, and penetrate through the extracellular matrices surrounding the oocyte [84]. Sperm meet the oocyte cumulus complex in the ampulla where fertilization normally occurs, and after fertilization, the preimplantation embryo undergoes cleavage as it is transported through the ampulla and the isthmus to the uterus for implantation [47]. Movement through the ampulla may involve both ciliary beating and smooth muscle contraction. When sections of the ampulla were surgically reversed in their orientation, few rabbits became pregnant [85]. In cases where pregnancy did occur, muscle contraction apparently overcame ciliary beating toward the ovary, showing that the cilia in the ampulla normally play an important role in controlling movement into the isthmus [85]. The isthmus of the oviduct is essential for normal reproduction, as its removal results in infertility [86].

### (2) Transport of preimplantation embryos to the uterus

A number of factors can influence the transport of preimplantation embryos through the ampulla and isthmus of the oviduct. Interestingly, the oviduct can distinguish between unfertilized oocytes and preimplantation embryos. which are transported at different rates, with embryos reaching the uterus one day earlier than unfertilized oocytes [87]. The production by embryos of platelet-activating factor (PAF), which mediates signaling to the oviduct, accelerates the passage of preimplantation embryos, but not oocytes, through the oviduct [88]. PAF may affect transport by increasing ciliary beating [89]. Human embryos likewise release PAF *in vitro*, and human oviducts synthesize both the PAF receptor and PAF acetylhydrolase, which degrades PAF, further supporting a role for PAF in the embryo-oviductal dialogue [90]. When rat embryos of different ages were transferred to the oviduct of pregnant females, older embryos reached the uterus before younger ones, again suggesting differential transport rates of embryos that depend on age [91]. These data from hamsters and rats support the idea that embryo transport is at least, in part, subtly controlled by the embryos themselves. Other factors such as maternal age and parity also influence embryo transport [92]. In hamsters, transport to the uterus occurred faster in young nulliparous females than in nulliparous or multiparous adult females. In the group of young females, but not the adults, development of the embryos was also highly synchronous.

Transport of preimplantation embryos through the oviduct is accomplished by smooth muscle contraction and ciliary beating [76,93]. However, the relative contributions of these two processes are not yet completely understood, and it is probable that both play roles in transport. The ampulla and isthmus are both lined by ciliated cells, which beat in the direction of the uterus [76]. The relative number of cilia decreases and the thickness of the muscle layers increases proceeding toward the uterine end [94], suggesting that cilia become relatively less important in the isthmus. Muscle contraction produces oscillating movements in the isthmus [64,95,96] that result in a net transport of preimplantation embryos towards the uterus [97]. Nitric oxide synthase inhibitors increased muscular activity in rats, and this was accompanied by increased rate of movement of eggs or microspheres in the oviduct [97]. These data support the idea that that muscle contraction can modulate (speed or slow) transport through the oviduct. Muscle contractions may also be important in keeping embryos grouped together as they are transported through the isthmus [98].

Muscle contraction in the oviduct is regulated by a variety of factors; however, the interplay of these factors with each other is not yet well understood. The oviductal muscles are innervated by the sympathetic nervous system [99,100]. Stimulation of α adrenergic receptors promotes contraction of the oviductal muscles, while stimulation of β receptors inhibits contractions [100-102]. Alternating contraction and relaxation produce the oscillatory movements involved in embryo transport. However, the adrenergic neurons may not be the primary means for controlling embryo transport since experimental depletion or inhibition these neurons does not prevent transport nor decrease fertility [35]. Chemicals produced by the oviduct itself or the preimplantation embryo can also modulate muscle contraction and may play the lead role in embryo transport. Oviductal muscle responds to sex steroids and prostaglandins. Endogenous estrogens stimulate oviductal muscle contraction, while progesterone relaxes it [103]. Likewise the prostaglandins PGF and PGE contract and relax oviductal muscle respectively [104-106]. Human oviduct smooth muscle also produces the prostaglandin prostacyclin which decreases muscle contractility and may affect embryo transport [107]. Oviductal smooth muscle also contains a nitric oxide system [108] that promotes muscle relaxation [109]. Inhibition of nitric oxide syntheses in rats increases oviductal motility and results in accelerated movement of embryos through the oviduct [97]. In additions to prostaglandins, the oviduct produces, endothelin-1 [110] and angiotensin II [111] which are involved in modulating oviductal muscle contraction and regulation of embryo transport. Recent data from the cow suggest that tumor necrosis factorα from the oviductal epithelium, immune cells of the oviduct, or even the embryo itself stimulates the release of these effectors from the oviductal epithelial cells [111]. A newly uncovered transport regulatory mechanism involves the cannabinoid receptor CB1 [112]. When CB1 is genetically or pharmacologically silenced, embryos are retained in the oviduct. This effect can be reversed by isoproterenol, a β adrenergic agonist. These data suggest that cannabinoid signaling is important in coordinating oviductal muscle contraction, and is necessary for proper embryo transport. While this review deals with conventional cigarette exposure, these results with the CB1 receptor suggest that exposure to marijuana for either recreational use or pain relief could affect embryo transport and hence female fertility. It is clear from the preceding that the regulation of embryo transport through the oviduct is complex and may depend on multiple regulatory mechanisms, some of which are just now being identified.

### (3) Biological importance of pick-up and transport by the oviduct

Timing of oocyte pick-up and embryo transport is critical as the preimplantation embryos must arrive in the uterus during the window when implantation can occur [113,114]. If the oocyte is not picked up by the oviduct or if the embryo moves through the oviduct too quickly or too slowly, implantation may fail to occur or may be ectopic. In rats, embryo transport was accelerated by treatment with methoxychlor, an estrogenic pesticide, and the embryos failed to implant in the uterus [115]. Likewise women treated with ergonovine maleate, a powerful stimulant of oviductal contraction, showed decreased conception rates when the drug was delivered immediately post coitus [116]. Interference with embryo transport can adversely affect fertility and in humans lead to ectopic implantation.

## C. Evidence that the oviduct is a target of cigarette smoke

While epidemiological studies have been clear in identifying increased reproductive risks for women who smoke both actively and passively (Section A), the reasons that smoke causes reproductive problems are usually not understood. Some of the risk factors for women smokers, such as ectopic pregnancy, failure to conceive, increased time to conception, could be due to effects of smoke on the pick-up and transport by the oviduct. We will next examine the *in vivo *and *in vitro *evidence supporting the idea that the oviduct is targeted by cigarette smoke.

### (1) In vivo evidence that the oviduct is a target of smoke

Direct inhalation of whole smoke has been shown in several studies to adversely affect the oviduct. Oviductal motility is altered in humans [117] and in rabbits [118] by inhalation of mainstream smoke. Inhalation of mainstream or sidestream smoke by hamsters, at serum cotinine levels that were within the ranges found in active and passive human smokers (mainstream = 72.8 and sidestream = 14.9 ng cotinine/mL) produced blebbing of the oviductal epithelium at the ultrastructural level and decreased the ratio of ciliated to secretory cells in the ampulla [119]. In a related study on hamsters, inhalation of either mainstream or sidestream smoke at levels that produced serum cotinine levels equivalent to those in human smokers (mainstream = 50–250 and sidestream = 18–80 ng cotinine/ml) slowed preimplantation embryo transport through the oviduct [120]. In addition, muscle contractions of the ampulla were significantly inhibited *in vivo *during smoke exposure, supporting the conclusion that embryo transport rates were slowed by inhibition of smooth muscle contraction [120]. While smooth muscle contraction rates did increase after smoke exposure stopped, they did not return to control levels, showing that inhibition of contraction by smoke is not immediately completely reversible.

Several *in vivo *studies using animal models have established that the oviduct is a target of nicotine, a major constituent of cigarette smoke. When administered to mice in drinking water, nicotine (108 μmol/L) significantly decreased Na and K ion concentrations in the oviductal epithelium [121]. In addition, nicotine injected subcutanenously (2.5 mg twice daily) into rats produced a significant increase in lactate dehydrogenase levels in flushings of the oviduct in early pregnancy [122]. While a change in the ionic composition of the oviductal epithelium or its secretions might adversely affect adhesion of the oocyte to the oviductal surface and the oocyte pick-up process, the relationship between these nicotine-induced changes and oviductal functioning has not yet been established experimentally. Nevertheless, these studies do demonstrate that nicotine exerts effects on the oviductal epithelium.

Two additional studies on rats indicate further effects of nicotine on oviductal functioning. When pregnant rats were treated with pharmacological doses of nicotine (2.5 mg injected subcutaneously twice daily), preimplantation embryo transport was inhibited [123]. In addition, nicotine (5 mg/kg), when injected subcutaneously twice daily in pregnant rats, both retarded embryonic development and reduced blood flow to the oviduct [124]. Reduction of oviductal blood flow decreases smooth muscle contraction, which in turn can delay embryo transport [124,125]. In other studies, nicotine slowed oviductal contraction *in vivo *in the Rhesus monkey, which may prevent implantation [126]. Oral nicotine administration through drinking water (108 μmol/L) also interfered with oocyte maturation, fertilization, and early pregnancy in mice [121]. Collectively these data show that nicotine affects the composition and secretions of the oviductal epithelium, adversely affects preimplantation development, retards movement of embryos through the oviduct, and reduces blood flow to this organ.

In a study involving gamete intrafallopian transfer (GIFT), no differences were found among active, passive and non-smokers in number of oocytes retrieved; however the number of live births after GIFT was significantly lower for active smokers (10.5%) than for passive smokers (23.1%) or non-smokers (33.3%) smokers [127], which could indicate an effect of smoke on the human oviduct.

Taken together these *in vivo *studies demonstrate that the oviduct responds to exposure to both whole mainstream and sidestream smoke and to nicotine and that the transport of preimplantation embryos can be inhibited by cigarette smoking, apparently by an inhibition of oviductal smooth muscle contraction. *In vivo *studies have not yet been undertaken to determine if oocyte cumulus complex pick-up is slowed in smoke exposed animal models or humans.

### (2) In vitro evidence that smoke affects oviductal functioning

**Figure 6:**
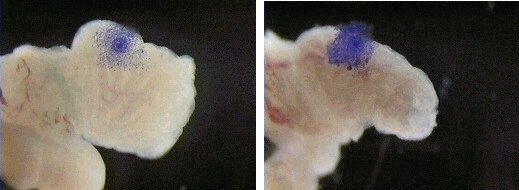
Micrographs showing oocyte cumulus complex pick-up by a hamster infundibulum of a control (6B) and a sidestream smoke treated (6A) preparation. Click the link to view a video of this experiment. During approximately 10 seconds of observation, the control complex moves toward the ostium while the smoked treated complex does not move. Reprinted from Molec Biol Cell 10:5–9, 1999 (with permission). See also

*In vitro *models have facilitated studies on smoke's effect on the oviduct and have further supported the conclusion that the oviduct is responsive to chemicals in cigarette smoke (Fig. 6, additional file 2). A hamster infundibular explant model [71] has been used to simultaneously measure ciliary beat frequency, adhesion, oocyte pick-up rate, and muscle contraction, before, during, and after exposure to smoke or its constituents [70,128-133]. In general, *in vitro *studies show that mainstream and sidestream smoke solutions adversely affect proper functioning of the oviduct. Mainstream and sidestream smoke solutions made from University of Kentucky 2R1 research cigarettes in a medium lacking bovine serum albumin (BSA) inhibited ciliary beat frequency in a dose dependent manner [128]. When BSA was included in the medium, mainstream solutions continued to inhibit beat frequency, while sidestream solutions either had no effect or slightly stimulated beat frequency, suggesting that the presence of BSA influenced how sidestream smoke affected beat frequency [129]. Interestingly, in both mainstream and sidestream solutions containing BSA, oocyte pickup rate was inhibited in a dose-dependent manner with sidestream smoke often being more inhibitory than mainstream smoke (Fig. 6). Since ciliary beat frequency was either not affected or slightly stimulated in sidestream smoke, these data show that smoke can inhibit oocyte pick-up rate by affecting factor(s) other than ciliary beat frequency. Oocyte pickup rate was more sensitive to the gas than the particulate phase of mainstream and sidestream smoke solutions [129].

Since pick-up rate decreased in sidestream smoke when beat frequency increased, oocyte pick-up rate must depend on factor(s) other than ciliary beat frequency [129]. Since adhesion of the oocyte cumulus complex to the tips of the cilia is important in oocyte pick-up [66,68,72], the effect of smoke solutions on adhesion was measured *in vitro *using the hamster infundibular model. Both mainstream and sidestream solutions inhibited oocyte cumulus complex pick-up rate and increased adhesion of the cumulus to the oviduct [133]. As was shown previously using wheat germ agglutinin [72], increasing adhesion by exposure to smoke leads to a decrease in pick-up rate since the complex can not be moved by the cilia if it adheres too tightly to the oviduct. These effects on adhesion and pick-up rate were observed when only the oocyte cumulus complex was pretreated with smoke solution or when only the infundibulum was pretreated, indicating that both the cumulus matrix and oviduct are targets of smoke treatment [133]. The oviduct was more sensitive to treatment than the oocyte cumulus complex, perhaps because smoke pretreatment affected both ciliary beat frequency and adhesion of infundibula but only adhesion of oocyte cumulus complexes. These data indicate that factors that increase adhesion of the oocyte cumulus complex to the cilia can decrease pick-up rate and explain why both mainstream and sidestream smoke solutions decrease pick-up rate even when ciliary beat frequency is increased by treatment with sidestream smoke.

The above studies were all done using non-filtered 2R1 research brand cigarettes manufactured at the University of Kentucky. A subsequent study examined the effects on oviductal functioning of smoke solutions from a filtered research brand cigarette (1R4F), various traditional filtered and non-filtered commercial cigarettes (Marlboro Red, Marlboro Light, Camel filtered, Camel unfiltered, Kool, and Kool with the filter removed), and three brands of harm reduction cigarettes (Advance, Omni and Omni Light) [134]. Harm reduction cigarettes have recently been introduced commercially and are claimed to contain fewer carcinogens than traditional commercial brands [135]. All of the cigarettes tested (research, traditional commercial, harm reduction) adversely affected oviductal functioning, and the effects were in general stronger on oocyte pick-up rate and smooth muscle contraction than on ciliary beating. Sidestream smoke generally produced a stronger effect in all assays than mainstream smoke solution. Smoke from the 1R4F cigarettes, which more closely resemble the commercial brands smoked today than the 2R1 cigarettes, was considerably more inhibitory in the pick-up rate and muscle contraction assays than the 2R1s. Except for mainstream smoke from Marlboro Lights and Kools, all traditional brand smoke solutions reduced pick-up rate by more than 60%. Except for mainstream smoke from Marlboro Lights and Camel filtered, all smoke solutions from traditional brands reduced muscle contraction by more than 80%. Smoke from harm reduction cigarettes reduced pick-up rate by 50–80% and reduced muscle contraction by 30–98% depending on the type of smoke and brand. These data show that the adverse effects observed on oviducts with 2R1 research cigarettes are also produced by filtered research cigarettes and by filtered, non-filtered and light commercial brands. Moreover, harm reduction cigarettes, while reduced in levels of carcinogens, still contain toxicants that can impair oviductal functioning.

Pick-up rate could also be altered by action of smoke on the oocyte cumulus complex, in particular the matrix which is required for adhesion to the cilia [72]. Both mainstream and sidestream smoke solutions from 2R1 cigarettes caused more dispersal of hamster cumulus cells during *in vitro *incubation than control medium lacking smoke, and oocyte pick-up rate was slowed when oocyte cumulus complexes were pretreated with smoke prior to measuring pick-up rate [133]. In addition,*in vitro *exposure of porcine oocyte cumulus complexes to nicotine, cadmium, and anabasine, all components of cigarette smoke, suppressed FSH induced expansion of the cumulus and decreased synthesis and accumulation of hyaluronic acid in the cumulus matrix [136]. These studies show that the matrix of the oocyte cumulus complex is also a target of cigarette smoke and damage to the matrix can affect pick-up of complexes by the oviduct.

## D. What chemicals in cigarette smoke impair oviductal functioning?

### (1) Chemicals most studied in smoke

Cigarette smoke is a complex suspension that contains over 4,000 chemicals distributed between a gaseous and particulate phase [17]. Most studies on cigarette smoke and its components have focused on nicotine [137-139], carcinogens [140,141], polycyclic aromatic hydrocarbons (PAHs) [142-144], such as benzo-a-pyrene, tobacco-specific nitrosamines [145,146], carbon monoxide [147,148], tar [149-151], metals (cadmium, lead) [152,153], and ten to fifteen other compounds including, phenol, acrolein, acetaldehyde, hydrogen cyanide, and formaldehyde [17,154-164]. Most of these chemicals have been linked to cancer or cardiovascular and lung disease. Of the 4,000 compounds found in cigarette smoke, at least 50 are known carcinogens [141]. PAHs and tobacco-specific N-nitrosamines are major contributors to lung cancer [154,161,165,166], while tar and carbon monoxide are major contributors to cardiovascular disease and chronic obstructive lung diseases [161,163,167]. PAHs have also been shown to initiate or promote atherosclerosis in avian [168-170] and mammalian [171] models. The PAH benzo(a)pyrene, induces atherosclerosis by stimulating proliferation of vascular smooth muscle cells that migrate into the vessel lumen [172,173].

### (2) Chemicals in smoke that affect the oviduct

Most of the above-mentioned chemicals that are commonly studied in smoke have not been studied with respect to their effects on the oviduct. An exception is nicotine, which did alter oviductal epithelium secretion and ion composition, embryo transport, embryo development, and oviductal blood flow in several *in vivo *studies (Section C1) and cumulus expansion *in vitro *[136].

#### (a) Ciliotoxic chemicals

Numerous studies have shown that cigarette smoke contains chemicals that are toxic to cilia of the mammalian respiratory system [174-178], the amphibian palate [179], *Paramecium *[180,181], and the gills of mussels, clams, and mollusks [182,183]. Moreover, nicotine increased ciliary beat frequency in the ferret trachea [184], formaldehyde inhibited respiratory cilia in the rabbit and pig [185]; and hydrogen cyanide, acrolein, and acetaldehyde inhibited ciliary beating in the clam [182]. Using an *in vitro *infundibular bioassay, the individual smoke constituents, which had been previously shown to be ciliotoxic in non-oviductal systems [175,176,182], were tested specifically for their effect on oviductal cilia [186]. Potassium cyanide (KCN), acrolein, phenol, acetaldehyde, and formaldehyde all inhibited ciliary beat frequency in a dose dependent manner *in vitro *[186]. However, only KCN was present in cigarette smoke solutions in a high enough concentration to account for the effect seen *in vitro*. KCN also inhibited oocyte pick-up rate. Nicotine did not inhibit ciliary beat frequency of the hamster oviduct, except at extremely high doses (Talbot, unpublished data).

#### (b) Pyridines, pyrazines, and phenols

Since cigarette smoke contains over 4000 compounds [17], there are likely other chemicals present in mainstream and sidestream cigarette smoke that can adversely affect oviductal functioning. To identify such chemicals, mainstream smoke solution from 2R1 cigarettes was fractionated by passage through 12 different solid phase extraction cartridges [77]. The eluates from each cartridge were screened using the hamster infundibular bioassay, and three cartridges were identified that retained inhibitory activity in the ciliary beat frequency, oocyte pick-up rate, and smooth muscle contraction bioassays. The chemicals in the eluates of each cartridge were then identified using GC-MS, and authentic standards of the identified compounds were purchased from commercial sources and tested independently to determine their activity in each of the three infundibular bioassays.

Pyridines, pyrazines, and phenols were the three major groups of chemicals identified in the inhibitory eluates (Table 1) [77,131,132]. Several other types of compounds including quinolines, indoles, and cyclopenten-1-ones were also present [132]. Within all groups, chemicals were identified that were highly inhibitory in all three bioassays, and some of the chemicals had LOAELs (lowest observed adverse effect levels) in the nano, pico and femtomolar range (Table 1). In general, if a chemical were inhibitory, it acted in all three bioassays, although the potency and efficacy for a particular chemical varied among the assays. Some of the compounds that were identified in this screen (Table 1) were previously thought to be safe and are included on the FEMA GRAS list (Flavor and Extract Manufacturers' Association – Generally Regarded As Safe) and the FDA EAFUS list (Everything Added to Food in the United States). Some of these chemicals are added to tobacco to flavor it (Table 1). For example, 3-ethylpyridine, which was inhibitory in all three bioassays at picomolar doses, is on the list of 599 chemicals added to tobacco in the United States [187]. Of the seven pyrazines tested, six are on the FDA EAFUS list, and in all but three assays, the pyrazines had LOAELs in the nanomolar or picomolar range. Indole and isoquinoline were the most toxic of all chemicals tested with LOAELs in the femtomolar range, except for isoquinoline which had a picomolar LOAEL in the ciliary beat frequency assay.

**Table 1:** Chemicals in Cigarette Smoke that Impair Oviductal Functioning ^1^

**LOAELs (M)**^2^						
**CHEMICALS**	**Oocyte**	**Ciliary Beat**	**Contraction**	**FEMA**	**FDA**	**ADDED**^5^

	**Pick-up Rate**	**Frequency**	**Rate**	**GRAS**^3^	**EAFUS**^4^	

PYRIDINES						
2-ethylpyridine	9.35 × 10^-12^	9.35 × 10^-12^	9.35 × 10^-12^			
4-methylpyridine	9.50 × 10^-11^	9.50 × 10^-11^	9.50 × 10^-11^			
2-methylpyridine	9.35 × 10^-11^	9.35 × 10^-11^	9.35 × 10^-10^			
4-ethenylpyridine	9.30 × 10^-11^	9.30 × 10^-9^	9.30 × 10^-11^			
3-ethylpyridine	9.33 × 10^-10^	9.33 × 10^-11^	9.33 × 10^-10^	√	√	√
Nornicotine	6.85 × 10^-9^	6.85 × 10^-8^	6.85 × 10^-8^			
beta-nicotyrine	6.33 × 10^-9^	6.30 × 10^-8^	X^6^			
2,4,6-trimethylpyridine	8.25 × 10^-8^	8.25 × 10^-6^	8.25 × 10^-8^			
2,4-dimethylpyridine	9.34 × 10^-7^	X^6^	9.34 × 10^-9^			
2,3-dimethylpyridine	9.34 × 10^-7^	9.34 × 10^-7^	X^6^			
4,4-bipyridine	8.78 × 10^-6^	8.78 × 10^-7^	8.78 × 10^-4^			
2,5-dimethylpyridine	9.34 × 10^-5^	X^6^	9.34 × 10^-5^			
3,4-dimethylpyridine	1.76 × 10^-5^	X^6^	1.76 × 10^-4^			
pyridine	1.27 × 10^-5^	1.27 × 10^-3^	1.27 × 10^-3^	√	√	√
3-methylpyridine	1.23 × 10^-5^	X^d^	1.23 × 10^-2^			
2,2-bipyridine	8.74 × 10^-4^	8.74 × 10^-2^	8.74 × 10^-2^			
cotinine	2.84 × 10^-2^	2.84 × 10^-5^	X^6^			
nicotine	9.01 × 10^-2^	X^6^	6.70 × 10^-2^			
						
PYRAZINES						
2-methoxy-3-methylpyrazine	10^-12^	10^-9^	10^-12^			
pyrazine	10^-11^	10^-12^	10^-9^	√	√	
2-methylpyrazine	10^-11^	10^-12^	10^-11^	√	√	√
2-ethylpyrazine	10^-11^	10^-12^	10^-12^		√	
2,5-dimethylpyrazine	10^-11^	10^-8^	10^-9^	√	√	√
2,3,5-trimethylpyrazine	10^-10^	10^-9^	10^-9^	√	√	√
2,6-dimethylpyrazine	10^-9^	10^-6^	10^-7^	√	√	√
						
PHENOLS, INDOLES, OTHERS						
Indole	10^-14^	10^-13^	10^-15^		√	
Isoquinoline	10^-13^	10^-12^	10^-13^		√	
4-Ethylphenol	10^-12^	10^-11^	10^-12^	√	√	
Quinoline	10^-11^	10^-13^	10^-11^		√	
4-Methylphenol	10^-11^	10^-12^	10^-11^	√	√	
2-Methylphenol	10^-11^	10^-9^	10^-11^	√	√	
5-Methylindole	10^-11^	10^-10^	10^-10^			
2,6-Dimethoxyphenol	10^-11^	10^-10^	10^-9^		√	
Hydroquinone	10^-10^	10^-10^	10^-10^			
3-Methyl-2-cylcopenten-1-one	10^-10^	10^-7^	10^-10^			
2,4-Dimethylphenol	10^-10^	10^-9^	10^-9^			
2-Methoxyphenol	10^-10^	10^-8^	10^-8^			
2-Cyclopenten-1-one	10^-9^	10^-7^	10^-9^			
4-Methoxyphenol	10^-8^	10^-7^	10^-7^			
2-Ethylphenol	10^-8^	10^-5^	10^-7^			
2,5-Dimethylphenol	10^-7^	10^-8^	10^-6^			
Benzene	10^-7^	10^-8^	10^-6^			
Phenol	10^-2^	10^-1^	10^-2^		√	

^1^Compiled from References 131, 132, and 135.

^2^LOAEL = Lowest observed adverse effect level in the oocyte pick-up rate, ciliary beat frequency, and muscle contractions assays.

^3^Chemicals known to be on the FEMA GRAS list. Others may also be on this list.

^4^Chemicals on the FDA EAFUS list (Everything Added to Food in the United States).

^5^Chemicals that are added to cigarettes by American tobacco companies.

^6^No effect at the highest dose tested.

Many of the compounds in Table 1 were also screened using a chick chorioallantoic membrane (CAM) assay that measures growth of the CAM and chick embryo [188,189]. In the CAM assay, many pyridines and pyrazines inhibited CAM growth dramatically, even at very low doses, and in some cases they also inhibited embryo growth. It is interesting that the chemicals in Table 1 were inhibitory in assays that measure diverse biological processes (ciliary beat frequency, oocyte pick-up, smooth muscle contraction, growth). It is not yet known if inhibition occurs by distinct mechanisms or if a basic underlying mechanism, such as inhibition of ATP production, was affected. Nor is it known if the chemicals act directly or indirectly, but given the extremely low effective doses of some of the chemicals, activation of a signaling cascade is possible.

These data from the oviduct and CAM studies indicate a need for further toxicological testing on the chemicals in Table 1, especially since some of them are used routinely in consumer products including food, cigarettes and cosmetics. These data also indicate a need for additional studies on the chemicals in smoke in general. The solid phase cartridge screen found about 40 oviductal toxicants, most of which were not previously recognized as smoke toxicants. It is probable that there are other toxicants in cigarette smoke that have not yet been identified as harmful nor studied in detail.

## E. Concentrations of smoke toxicants in cigarettes and in human smokers

The data on oviductal toxicants beg the question – what are the concentrations of these compounds in cigarette smoke and in actual active and passive smokers? Some of the oviductal toxicants, such as nicotine, have been studied extensively, and concentrations are well documented in both cigarettes and smokers [17,190-192]. The LOAEL concentrations of 2-ethylpyridine, 2-methylpyridine, and 3-ethylpyridine are about 10,000 to a million times lower than the concentration of these chemicals in mainstream and sidestream smoke from commercial cigarettes and cigars [77,193]. However, some of these toxicants, such as 3-ethylpyridine, have not previously been recognized as harmful, and little is known about their concentrations, in smokers. Many chemicals were inhibitory in the infundibular bioassays at nano and picomolar doses suggesting that they could be effective *in vivo *at extremely low doses that would be difficult to detect and measure. Concentrations of cigarette smoke components, such as phenolic compounds, vary among different brands of cigarettes [194]. For example, different types of cigarettes, such as Indian Bidi cigarettes, have higher concentrations of phenols than traditional commercial cigarettes [157]. Concentrations of chemicals also vary between various research brand cigarettes [195]. Finally, the source of the smoke also affects chemical concentration. While mainstream and sidestream smoke have similar chemicals the relative amounts of particular chemicals can vary significantly between the two types of smoke [17].

Cigarette smoke chemicals or their metabolites must gain access to the circulatory system and reach their target organs to exert their toxicity. Studies have measured nicotine, cotinine (a metabolite of nicotine), and other cigarette smoke components in reproductive tissues, although little is known about concentrations in the oviduct *per se*. Interestingly, levels of cigarette toxicants in reproductive tissues or fluids can be significantly higher than in serum. For example, pregnant rabbits injected with tritiated nicotine had 5–11 times higher nicotine concentrations in uterine fluid than in plasma [196]. Both nicotine [197] and phenols [198] have been detected in the cervical fluid of smokers, and nicotine concentrations in the cervical fluid (66–2620 ng/ml) were significantly higher than in serum (0.1–39 ng/ml) [199].

Cigarette smoke components have also been detected in the follicular fluid of smokers [200,201]. Cotinine, a biomarker for smoke exposure, was higher in the follicular fluid of active smokers (mean = 285.69 ng/ml; range = 62.21–595.00) than passive smokers (mean = 29.65 ng/ml; range = 20.91–45.75) and nonsmokers (mean = 3.71 ng/ml; range = 1.20–15.62) [200]. Cadmium, like other smoke components, does reach and accumulate in the follicular fluid of smokers (7.93 ng/mL) [202], and cadmium concentration in the ovary was elevated in smokers (150 ng/g) versus nonsmokers (115 ng/g) [203]. While cadmium apparently does not interfere with embryo transport through the oviduct in the rat [204], it is another example of a smoke toxicant that can accumulate in reproductive organs and which, in laboratory animals, can adversely affect reproduction [205]. While many of the toxicants that affect the oviduct have not been quantified in smokers and there is little known about their concentrations in the oviduct, those chemicals that have been studied, appear to reach the reproductive organs and are often found in higher concentration in reproductive tissues and fluids than in serum or urine.

## F. Summary

The oviduct, while seemingly a simple organ, is exquisitely designed to convey gametes in opposite directions virtually simultaneously and to provide a suitable environment for preimplantation development and transport of embryos to the uterus for implantation. It is vital for reproductive success. Factors that interfere with its functioning can adversely affect fertility. The oviduct serves as a useful model to evaluate the effect of cigarette smoke and its components on a reproductive organ and in a more general sense on a variety of biological functions. While most work on smoke's effect on the oviduct has been done on ciliary beat frequency, oocyte pick-up rate, cilia-oocyte cumulus complex adhesion, and smooth muscle contraction, other parameters of oviductal functioning could be added to this array of bioassays. For example, monitoring the synthesis and secretion of the oviductal proteins may give further insight into how smoke affects oviductal functioning and secretory processes in general. The oviductal assays have been useful in identifying numerous smoke toxicants many of which were not previously recognized as harmful and some of which are widely used in consumer products. Further studies on the safety of these chemicals are needed. Commercial brands of cigarettes contain toxicants capable of shutting down oviductal functions *in vitro *and interestingly sidestream smoke is often more inhibitory than mainstream smoke. Harm reduction cigarettes, while apparently reduced in carcinogens, still contain chemicals that impair basic biological processes including ciliary beating, oocyte pick-up, and smooth muscle contraction. The effects of smoke on the heart and lungs is widely known and well documented. The effects of smoke on the oviduct, which are just recently becoming more widely recognized, demonstrate that organs remote from the site of inhalation may be adversely affected by chemicals in smoke are consistent with the idea that all organs are targets of smoke [1]. Active and passive smokers of reproductive age should be made aware of the possible dangers in smoking and how smoking could affect their reproductive ability.

## Supplementary Material

Additional File 1 Video movie showing a stained oocyte cumulus complex (blue) being pick-up by a hamster infundibulum. The complex adheres to the surface of the oviduct and is pulled along towards the ostium by ciliary beating. Reprinted from Molec Biol Cell 10:5–9, 1999 (with permission). Click here for file

Additional File 2 Video movies showing a control (right) and smoke treated infundibulum. The oocyte cumulus complex (blue) on the control oviduct moves over the surface of the infundibulum and is picked up at the normal rate. In the smoke exposed preparation, the oocyte cumulus complex barely moves during the same interval of time. Reprinted from Molec Biol Cell 10:5–9, 1999 (with permission). Click here for file
